# Enhanced multimodal biometric recognition systems based on deep learning and traditional methods in smart environments

**DOI:** 10.1371/journal.pone.0291084

**Published:** 2024-02-15

**Authors:** Sahar A. El_Rahman, Ala Saleh Alluhaidan

**Affiliations:** 1 Department of Electrical Engineering, Faculty of Engineering-Shoubra, Benha University, Cairo, Egypt; 2 Information Systems Department, College of Computer and Information Sciences, Princess Nourah Bint Abdulrahman University, Riyadh, Saudi Arabia; Firat Universitesi, TURKEY

## Abstract

In the field of data security, biometric security is a significant emerging concern. The multimodal biometrics system with enhanced accuracy and detection rate for smart environments is still a significant challenge. The fusion of an electrocardiogram (ECG) signal with a fingerprint is an effective multimodal recognition system. In this work, unimodal and multimodal biometric systems using Convolutional Neural Network (CNN) are conducted and compared with traditional methods using different levels of fusion of fingerprint and ECG signal. This study is concerned with the evaluation of the effectiveness of proposed parallel and sequential multimodal biometric systems with various feature extraction and classification methods. Additionally, the performance of unimodal biometrics of ECG and fingerprint utilizing deep learning and traditional classification technique is examined. The suggested biometric systems were evaluated utilizing ECG (MIT-BIH) and fingerprint (FVC2004) databases. Additional tests are conducted to examine the suggested models with:1) virtual dataset without augmentation (ODB) and 2) virtual dataset with augmentation (VDB). The findings show that the optimum performance of the parallel multimodal achieved 0.96 Area Under the ROC Curve (AUC) and sequential multimodal achieved 0.99 AUC, in comparison to unimodal biometrics which achieved 0.87 and 0.99 AUCs, for the fingerprint and ECG biometrics, respectively. The overall performance of the proposed multimodal biometrics outperformed unimodal biometrics using CNN. Moreover, the performance of the suggested CNN model for ECG signal and sequential multimodal system based on neural network outperformed other systems. Lastly, the performance of the proposed systems is compared with previously existing works.

## Introduction

Multimodal biometric recognition systems are the future of smart environments’ security [1]. The systems that use biometrics have different features over the traditional systems, which use pin-based authentication [1, 2]. Multimodal biometrics are executed at various fusion levels and fulfill greater identification performance than unimodal biometrics [3, 4]. Also, it provides anti-spoofing measurement, where the intruder will find it difficult to spoof multiple biometric modalities Electrocardiogram (ECG) information along with other biometrics will minimize credentials forgery and intrusion. Utilizing ECG as one of biometrics provides an advantage to the system where the heartbeat is inherited to a subject that is secured, confidential, and difficult to be forged. These days, fraud identification in smart environments is considered one of the most criminal security issues. To prohibit these issues, many methods are utilized like the techniques of biometrics identification, because of the necessity of extremely reliable security methods in confidential systems [5]. Biometric identification systems are highly used in several public security systems [6, 7] such as surveillance, recognition, law enforcement to attain superior recognition performance [8, 9]. In smart environments’ context, multimodal biometrics system has been employed to verify individuals and make verification simpler (Rajasekar et al., 2022). They can identify subjects depend on behavioral features, physical features [10], or physiological signals, such as ECG and EEG (Electroencephalogram) [6, 11]. Real-time recognition systems use the patterns and templates which are extracted from individuals and matched with enrolled ones [7] to decide for acceptance or rejection [8, 12, 13].

### Problem statement

There are some issues in real-time unimodal systems, like spoof attacks, sensed data artifacts, interclass variations, and interclass similarities [14, 15]. Multiple biometric sources are used now and needed for reliable identification. With advances in artificial intelligence more accurate results and better performance are achieved. Therefore, a multimodal biometric system is suggested to reduce some of these issues by the fusion of ECG and fingerprint modalities. ECG provides the benefit of liveness recognition to enhance biometric system robustness [14]. Studies of the combination of ECG with a fingerprint as multimodal biometric systems are few [15, 16] conducted different traditional classifiers on multimodal biometric systems based on different fusion levels of ECG and fingerprint. This study presents a study of the performance of fusing ECG and fingerprint biometric modalities with different classification techniques and different fusion levels.

### Contribution

To summarize, previous studies were more focused on one modality or one biometric for authenticity. Our approach increases the accuracy of classification by combining parallel and sequential multimodal biometric systems with various feature extraction and classification methods. The proposed multimodal systems of ECG with fingerprint modalities comparing the deep learning approaches with traditional methods become more proper and efficient to be applicable. The key contribution of this study can be briefly described as:

- Developing enhanced unimodal and multimodal biometric systems for smart environments based on CNN and traditional methods using different fusion levels of fingerprint and ECG signal.
- In comparison to other computational intelligence techniques, the proposed models can be used for recognition with satisfactory results and achieve improved rating optimization. A multimodal recognition system that combines an ECG signal with a fingerprint is effective.
- The efficiency of the proposed methodology is proved and the performance of the multimodal systems outperformed unimodal systems employing different classifiers, fusion levels, and rules.

The paper is organized as follows: Section 2 presents a literature review. Section 3 covers the details of the unimodal and multimodal biometrics methodology. Section 4 shows the results of the experiments. Section 5 has the conclusion.

## Related works

Various unimodal and multimodal biometric systems have been developed and presented by the researchers with the explanation of the success metrics of these systems.

### Fingerprint recognition system

Worldwide, fingerprint biometric is massively used by forensic experts and laboratories in criminal investigations, presence monitoring systems, and law enforcement systems [10, 17–19]. A fingerprint consists firstly of dark lines series that present the peaking and apparent part of the friction ridge skin, and secondly the white spaces that present the valleys between the ridges [20]. The ridge of a finger image is a curved line that is either a continuous line or ceases at a ridge ending. When 2-ridges intersect at a certain point, it is called a bifurcation. The bifurcation and ridge ending is considered as the minutiae points that are utilized to extract the unique features of fingerprints [10]. In fingerprint biometric systems, a feature extraction process is critical similar to the minutiae template matching [21].

Some researchers considered different methods such as neural networks, fuzzy logic, and deep learning to develop advanced robust fingerprint recognition techniques [18, 22, 23]. In [24] a fingerprint recognition system is suggested which utilizes wavelet transformation with simple minutiae equivalent to increase the reliability of fingerprint analysis and identification. Ali et al. [2] presented fingerprint identification and authentication method with minutiae equivalent approach with Euclidean distance. The tests are performed using two fingerprint databases. The research by Kahraman et al. [7]suggested fingerprint identification with image processing techniques. Multilayer perceptron method for features extraction and Regression Neural Network is applied for identification. Dale et al. [25] presented a system with the DCT features of a fingerprint while in Gupta & Walia [26] the suggested system with Gabor filter. Tico et al. [27] developed fingerprint recognition methods with diverse techniques such as Euclidian distance. Dale et al. [25], Borra et al. [8], and Mote [28] studied a classification technique of fingerprint with wave-atom transform for removing noise along with an adaptive genetic neural network for classification. Depending on the minutiae points matching or on the similarities in the fingerprint structure, there are many fingerprint systems are developed as listed in Table 1.

**Table 1:** Existed fingerprint biometric systems.

Researchers	Dataset	Techniques	Performance Metrics
[25]	FVC2000, FVC2002	Minutiae Matching Algorithm with Euclidean Distance	FVC2000: FAR = 0.2049, FRR = 0.1944, Acc = 80.03%
			FVC2002:FAR = 0.0154, FRR = 0.0137, Acc = 98.55%
[5]	8 scans, 6 fingers, 8 persons	Artificial Neural Network	Acc = 81%.
[6]	SLF, NIST SD27, FVC DB1, DB2	Artificial Neural network	Rank-1 Acc = 86.7%
[7]	UPEK, FVC2000	Regression Neural Network	Acc = 95.57% [UPEK)
			Acc = 91.38% (FVC2000)
[8]	FVC2000	Adaptive Genetic NN	Acc = 97.81%
[2]	FVC2000, FVC2002	Minutiae Matching Algorithm with Euclidean Distance	FVC2000: FAR = 0.2049, FRR = 0.1944, Acc = 80.03%
			FVC2002:FAR = 0.0154, FRR = 0.0137, Acc = 98.55%
[5]	8 scans, 6 fingers, 8 persons	Artificial Neural Network	Acc = 81%.
[6]	SLF, NIST SD27, FVC DB1, DB2	Artificial Neural network	Rank-1 Acc = 86.7%
[7]	UPEK, FVC2000	Regression Neural Network	Acc = 95.57% (UPEK)
			Acc = 91.38% (FVC2000)
[8]	FVC2000	Adaptive Genetic NN	Acc = 97.81%
[17]	FVC2004	FL	FL: AUC = 0.830
		LDA	NN: AUC = 0. 866
		NN	LDA: AUC = 0.832

### ECG recognition system

ECG can be employed solely for subjects’ identification or combined in a multimodal biometric recognition system [10]. It is considered a rising modality for the human identification system. The difference between the developed unimodal and multimodal systems is the extracting method of features and the features set characteristics [29]. ECG is used as a measurement of the heartbeat electrical activities [8]. It is considered one of the best biometrics since it represents the evidence of the individual’s aliveness [10]. The ECG is a physiological low-frequency signal [30] and includes repolarization and depolarization of the muscle fibers constructing the heart. The repolarization is corresponding to T-wave and the depolarization is corresponding to the QRS wave, P-Wave, and the other regions are considered as a baseline [31]. There are many ECG systems are developed as shown in Table 2. The motivation of using ECG is:

- The heartbeat signal of a person holds a unique signature and it is stable for a long time.
- The heartbeat signal offers direct solutions to the aliveness detection as it cannot be captured from deceased body parts, fake finger, or a high-resolution video. It is also difficult to steal and replicate a heartbeat signal. So the imposters will face a greater challenge to collect an illicit copy of the heartbeat signal of the actual user.

**Table 2:** Summary of the existing ECG biometric systems.

Researchers	Dataset	Techniques	Performance Metrics
[32]	50 Physionet dataset)	Signal Processing Techniques	Acc = 99%
[9]	100 TB dataset)	Haar wavelet transform	Acc = 97.12%
[10]	80 Physionet dataset)	DWT	Acc reaches 100%
		Random Forest	
[12]	13 (PTB dataset)	DCT and autocorrelation	94.47% (PTB dataset)
	13 MITBIH dataset)		97.8% (MI-TBIH dataset)
[14]	47 subjects (MIT-BIH dataset)	9-layer deep convolutional neural network	Augmented Data: ○ Acc = 93.47% with Noise: ○ Acc = 94.03% without Noise:Original Dataset: ○ Accuracy = 89.07% with Noise ○ Acc = 89.3% without Noise:
[15]	20 Subjects	SIMCA	Acc = 95.0%
[17]	20 Subjects	ED	AUC = 87.98347% (ED)
		FL	AUC = 89.98591% (FL)
		NMC	AUC = 78.98347% (NMC)
		LDA	AUC = 87.79034% (LDA)
		NN	AUC = 98.98591% (NN)
[18]	44 (MIT-BIH)	Support Vector Machine	Acc = 79.55% for (MIT-BIH)
			Acc = 84.9%, for (IIT (BHU)
	65 (IIT (BHU)	Features of Eigen beat + matching criterion of nearest neighbor.	Acc = 85.7% for (MIT-BIH)
			Acc = 92.49% for (IIT (BHU)
[20]	PhysioNet PTB Database	Artificial neural network	Acc = 94 .74%
		Naïve Bayes	
[33]	51 Subjects	J48 decision tree	Acc = 94 .40%
[17]	47 Subjects	FL	FL: AUC = 0.93688
		NN	NN: AUC = 0.951
		LDA	LDA: AUC = 0.90214

### Fusion level in identification

A multibiometric fusion method for identification using two modalities has been explored in research. For example, the iris and the fingerprint are processed to generate different score levels then fusion is applied on each score. Each score is split into three zones of interest. The fusion is applied using two approaches: classification and fuzzy logic. The proposed fusion methods outperform single modality approaches [34]. For a robust human identification system, the score level fusion of face and iris attributes are combined and re-classified to improve the individual unimodal systems performance. The result provide a proof on how accurate is the multimodal biometric system [35]. The paper by [36] suggests a methodology for combining the identification results of face and ECG data. The accuracy was 98.8%. “By using a fusion approach the identification accuracy improved to 99.8%” [36].

### Multimodal biometrics system

A single biometric modality method measures only one behavioral or physiological feature. So, it leads to a lower reliable system and can be impacted by the actual environmental conditions [6]. The multimodal biometric system reduces some of the issues using the acquired information from different sources [37]. The significance of biometric identification in smart environments is highlighted in (Rajasekar et al., 2022). The multimodal biometrics fusion can be implemented at various levels. The fusion levels are matching score, feature extraction, raw data, or decision level [7, 38–40]. The main frameworks of classification techniques combination are both, the first one; the same input modality presentation is utilized to all classifiers. In the second one, the different input modality presentation is considered for each classifier [41]. The fusion models are two categories that are sequential and parallel, where, parallel fusion provides higher accuracy [42]. In general, ECG is used as an auxiliary biometric in a multimodal biometric fusion that providing some advantages [43]. Where the fusion of many biometric modalities can improve the system performance and support anti-spoofing [6, 38]. Deep learning techniques are utilized in biometrics recognition to improve the system performance of the traditional classifiers. Several applications use traditional and deep learning techniques for biometric modalities. Gupta et al. [44] developed a new cancelable multimodal system that combines biometrics using the projection-based algorithm. The experiments are conducted by three chimeric multimodal datasets and the findings fulfill high performance. Hossain et al. [45] presented a multi-stage verification system utilizing multibiometrics and verifiers of multibiometrics. The researchers studied the symmetric rejection effectiveness for their proposed verification systems. Kant & Chaudhary [46] presented a multimodal biometric scheme that fusing the fingerprint, finger knuckles print, and palm print using score level. Arora et al. [47] implemented a CNN deep learning technique for combining the extracted features of the face and iris traits. Yudistira and Kurita [24] studied a multimodal CNN for multimodal correlations capturing over arbitrary time-stamps. The action recognition using a deep CNN was performed using spatial and temporal streams. The researchers developed a correlation network with a Shannon fusion approach by averaging the two streams to learn a pretrained CNN. This model was established to complement the existing approaches of network fusion [48]. Alay & Al-Baity [21] developed a multimodal identification system using three CNN deep learning models for human recognition with the modalities of face, iris, and finger vein. The researchers used CNNs for the features extraction and a softmax classifier is used for image classification. The system was developed by combining the three CNN models of the three modalities. Each CNN mode was built using the VGG-16 model, the optimization was applied using the loss function (categorical cross-entropy) and Adam algorithm. To prevent overfitting, image augmentation and dropout techniques were applied. Score and feature level fusion techniques were used for the CNN models fusion. There are different multimodal biometric systems established the traditional and the deep Learning techniques and using various fusion levels and modalities are developed. Most findings show that multimodal biometric systems outperformed unimodal biometric systems as indicated in Table 3.

**Table 3:** Summary of the existing multimodal biometric systems.

Researchers	Modalities	Sample Size + Dataset	Fusion Techniques	Performance Measurements
[21]	face, finger vein, and iris	SDUMLA-HMT dataset	CNN + score and feature levels	Acc = 99.39% with a feature level Acc = 100% with score level
[24]	spatial and temporal streams	UCF-101 + HMDB-51 datasets	CNN+ Shannon fusion approach	Acc = 94.2
[47]	Face and Iris	CASIA-Face V5 + IITD iris datasets	CNN	Findings proved the superiority of suggested multimodal system compared with unimodal one.
[26]	multimodal feature fusion	3-chimeric multimodal	projection-based	EER = 0.004
[44]	Face, Finger, and Iris	3-chimeric multimodal	Adaptive score fusion	Acc = 99.5%, EER = 0.5%
[45]	Fingerprint and face	517 Subjects	Score sets	Fingerprint: EER 0.0772 (L1) and 0.0547 (R1) Facse: EER 0.0463 (C) and 0.0579 (G) where LI, RI, C, and G are the score sets.
[49]	ECG and Palmprint	50 Subjects	Level of Matching Score	Acc = 82.1% for Palmprint Acc = 89% for ECG Acc = 94.7% for Multimodal
[50]	PCG and ECG	21 Subjects	Decision Level.	PCG: Rate of Identification = 88.7% for a certainty of 70%. ECG: Rate of Identification = 93% with 95% similarity threshold. Multimodal: Rate of Identification = 96.4%.
[51]	ECG and Fingerprint	Biopac is used for ECG signal, MP35 for different subjects	Score Generation	ECG: Matching Rate up to 69% when FAR = 2.38, FRR = 9.52 Fingerprint: Matching Rate up to 88.89% when FAR = 7.77, FRR = 5.55 Multimodal Biometric: Matching Rate up to 92.8% when FAR = 2.5, FRR = 0
[38]	Fingerprint, Face, and Hand Geometry	100 Subjects	Min-score Max-score Sum of scores	Fingerprint: Acceptance Rate nearly 83.6%Multibiometric: ○ Min-score: 85.6% ○ Max-score: 93.6% ○ Sum of scores: 98.6%
[42]	ECG and Fingerprint	45 Subject	SVM Weighted Sum Rule Likelihood Ratio	ECG: EER = 6.97% Fingerprint: EER = 2.22% Multimodal: EER = 1%
[52]	ECG, Face, and Fingerprint	50 Subjects	Weighted Sum Rule. Likelihood SVM	Multimodal outperforms unimodal biometrics system.
[53]	ECG, face, and fingerprint	78 Subjects	score fusion with Transformation	Multi-biometric system: EER = 0.22% ECG: EER = 10.80% Face: EER = 4.52% Finger: EER = 2.12%,.
[54]	Fingerprint and Face	200 Subjects	Score level	MOC(Finger1): EER = 0.062 MOC(Finger2): EER = 0.051 MOH(Face): EER = 0.021 Multimodal: EER = 0.007
[17]	ECG and fingerprint	47 Subjects	Score Level & Decision Level	Parallel+NN+Sum: 0.956 Parallel+NN+Max: 0.946 Parallel+NN+Product: 0.933 Parallel+FL+Sum: 0.945 Parallel+FL+Max: 0.925 Parallel+FL+Product: 0.901 Parallel+LDA+Sum: 0.917 Parallel+LDA+Max: 0.856 Parallel+LDA+Product: 0.888 Sequential+FL: 0.941 Sequential+NN: 0.985 Sequential+LDA:0.908

## Methodology

For setting experiment, data was prepared from multiple sources then augmented as detailed below. Models are explained in this section. We used Thinkpad Intel Core i3 with RAM 4GB and 256 GB hard disk and MATLAB for constructing CNN and performing the analysis.

### Dataset information

In many multimodal biometric systems, the databases of multiple biometrics are combined to generate virtual individuals instead of a gathering of the real biometrics from each individual [52]. In this study, a virtual database of multimodal biometrics is constructed to be conducted in the performance evaluation. It is gathered from two public databases of fingerprint (FVC2004) and ECG [54]. FVC 2004 (FVC2004) fingerprint database is utilized that contains four various datasets that contain 80 fingers each. The MIT-BIH [54] dataset is an ECG database that contains 47 subjects. The virtual multimodal dataset is generated using ECG and the fingerprint datasets for the performance evaluation. One individual from the ECG dataset is randomly allocated to an individual from the fingerprint dataset. The multi-modal dataset is gathered from 47 subjects, everyone has been assigned to two samples of ECG and fingerprint [16]. Extensive experimental testing is conducted for the evaluation of the proposed models with the original virtual dataset without augmentation (ODB) and the virtual dataset with augmentation (VDB).

A multimodal biometric system is a robust solution for several biometric recognition systems flaws. Integrating multiple biometrics is observed to be a general issue in diverse real biometric recognition systems. In this section, the performance of the suggested unimodal and multimodal recognition systems is assessed. The tests are performed on ECG and fingerprint datasets. The two databases used in the evaluation are MIT-BIH by Wang et al. [12] for ECG and FVC2004 (FVC2004) for fingerprint grounded on NN, FL, LDA classifiers. Extended experiments are implemented to assess the proposed system using 47 subjects’ data derived from a virtual multimodal database. Moreover, a ten-fold cross-validation technique is used for testing the recognition implementation. The following subsections describe average results. Denoting, in the first proposed parallel multimodal system, a score level fusion is used while in the second sequential multimodal system of ECG and fingerprint, a decision level fusion is applied. In this study, unimodal and multimodal biometric systems with CNN using different fusion levels of fingerprint and ECG signal are evaluated. More experiments are being conducted to evaluate the proposed models with ODB and VDB datasets.

#### Data augmentation

Data augmentation is utilized to develop a more robust model for overfitting. It is an approach to create an artificial dataset sample using the original ones [55]. It is utilized for increasing the size of training data and enhancing the performance [56, 57]. The augmentation implementation of the proposed dataset is based on the methods proposed in [56, 57]. Fingerprint database is augmented to construct a database by translations (two times), reflections (two times), and color manipulation (two times). The constructed dataset is six times greater than the original dataset. ECG dataset is augmented to construct a database by 15% scaling (two times) and 30% scaling (two times). The constructed dataset is four times greater than the original dataset.

### Biometric models based on traditional methods

#### ECG biometric model

**Fig 1:**
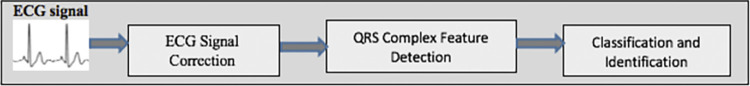
ECG biometric recognition model.

Fig 1 shows the model of ECG biometric. It is evaluated using MIT-BIH database [54] and the experimental testing is conducted on 47 subjects. The noise and artifacts in ECG signal are attenuated to be corrected by digital filters before QRS Complex feature detection. The QRS detection processing is achieved utilizing the Pan and Tompkins algorithm [58] which performs a competent process for extracting features of the QRS complex. The extracted features are entered into Linear Discriminant Analysis (LDA), Fuzzy Logic (FL), or Feature Map-Neural Network (SOM-NN) classifiers [17] to apply the features matching. Further details explaining the proposed technique are found in [16, 17].

#### Fingerprint biometric model

**Fig 2:**
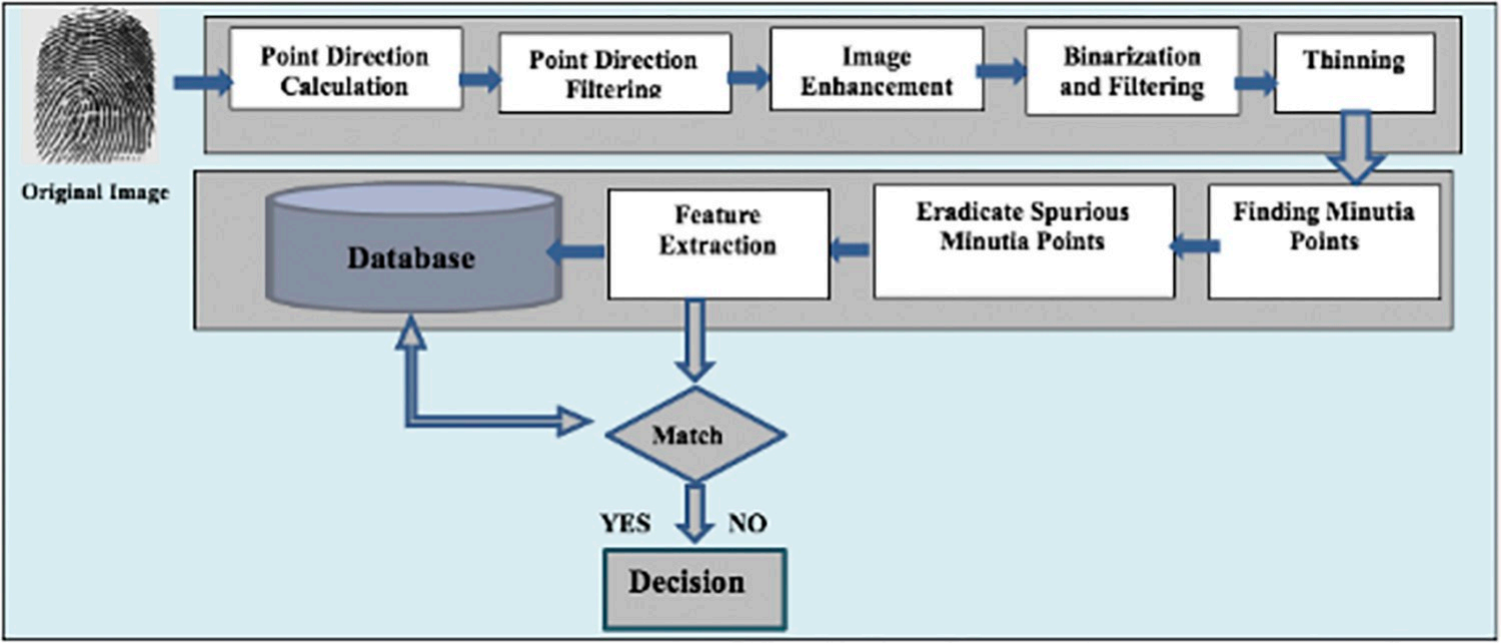
Fingerprint biometric model.

The fingerprint recognition model is indicated in Fig 2. The model consists of different phases, first phase is fingerprint image preprocessing, following phases are extraction of minutiae, detection of core point, then extracting features, and ultimately Identification. For a reliable recognition system, preprocessing of fingerprint images is the necessary phase before extracting the features and matching them. Preprocessing phase increases the clarity of ridge structure [12]. It is performed by Histogram Equalization (HE) and Fast Fourier Transform (FFT), then the preprocessed image is now available for subsequent processes. A very critical factor here is to have the right estimation of local ridge orientation during the extraction of minutia points and matching. The orientation map of the image is formed with a 3×3 Sobel filter [7]. Computationally, the Sobel filter is convolved with the image to calculate the derivatives using 2 kernels (3×3) (one for vertical and the second for horizontal changes). To calculate the oriented gradients Eq (1) and Eq (2) are utilized and Eq (3) is utilized to calculate all pixels directions.

$$G_{h}=[\begin{array}{ccc} -1 & -2 & -1\\ 0 & 0 & 0\\ 1 & 2 & 1 \end{array}]*A$$ (1)

$$G_{v}=[\begin{array}{ccc} -1 & 0 & 1\\ -2 & 0 & 2\\ -1 & 0 & 1 \end{array}]*A$$ (2)

$$\theta =\tan^{-1}\frac{G_{h}}{G_{v}}$$ (3)

where: A is the fingerprint image, ***G***_***h***_ is horizontal and ***G***_***v***_ is vertical derivatives, while ***θ*** is directions of all pixels.

**Fig 3:**
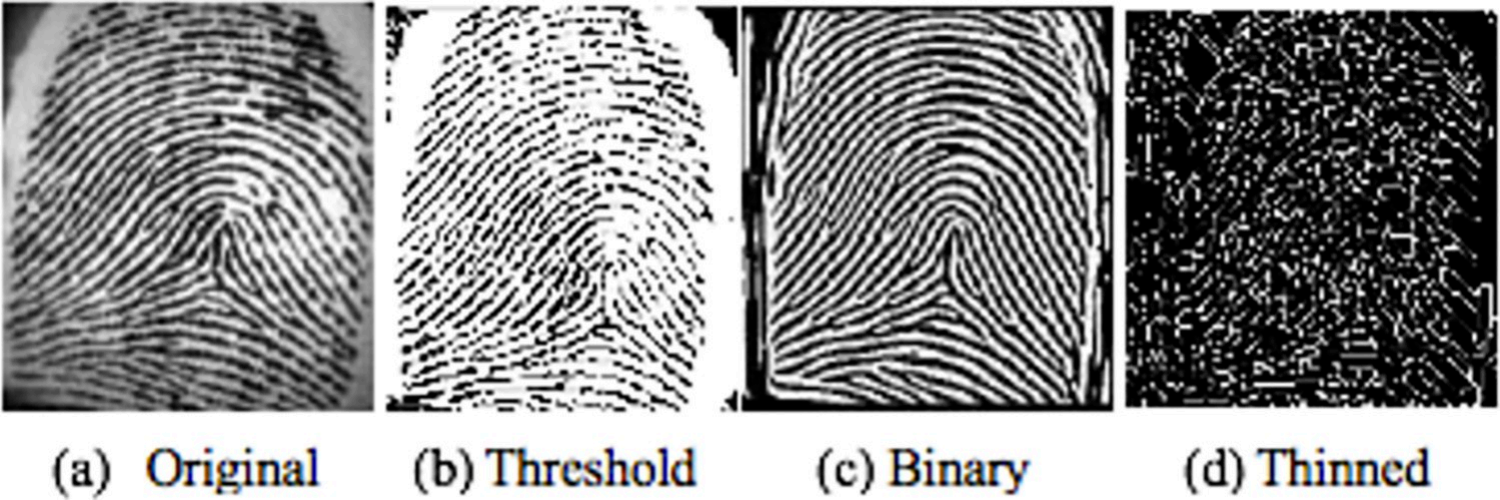
Preprocessing steps of fingerprint image.

**Fig 4:**
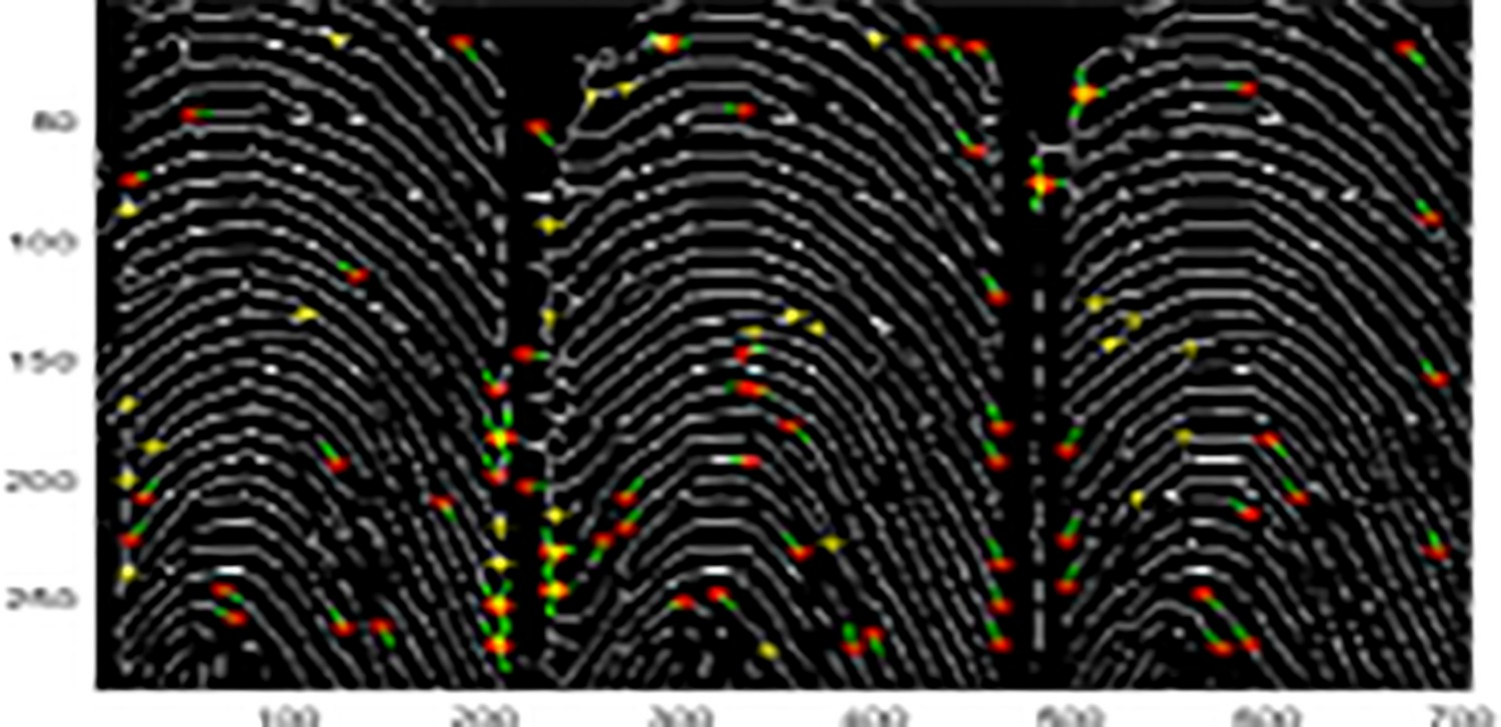
Sample for ridge ending and bifurcation.

Fig 3 indicates the preprocessing phases for the demonstrated sample image. The features are extracted after core point detection. The minutia point’s histograms are computed in a certain interesting region, which is considered as a vector of features. Then, a similar approach is applied to bifurcations and ridge endings. Fig 4 shows a sample for bifurcation and ridge ending combined. The feature vector length is 1460 which includes every feature extracted from images after they were filtered. This feature vector is entered into SOM-NN, LDA, or FL classifiers [16, 17] for the identification of fingerprint images. The model is evaluated with the FVC2004 database (FVC2004). More details about the proposed model are found in [16].

#### Fingerprint and ECG multimodal systems

In this study, two multimodal biometric systems are presented using fingerprint and ECG biometrics with different fusion levels. The fusion Levels are decision level and score level. The first system is a sequential multimodal system using the decision level fusion of fingerprint and ECG for human identification. The extraction of features is fulfilled, and then the biometric templates are constructed from these features. The extracted features are then fed into the matching stage. At this point, the NN, FL, and LDA classifiers are employed. Finally, the final decision is taken according to the decision level fusion. The second system is a parallel multimodal system using the score level fusion of fingerprint and ECG for human identification. When the extraction of features is fulfilled, the extracted features entered into the matching stage. The same classifiers used in the first system were used again at the matching stage. In the end, the final decision is taken according to the score level fusion.

### Sequential and parallel multimodal system

**Fig 5:**
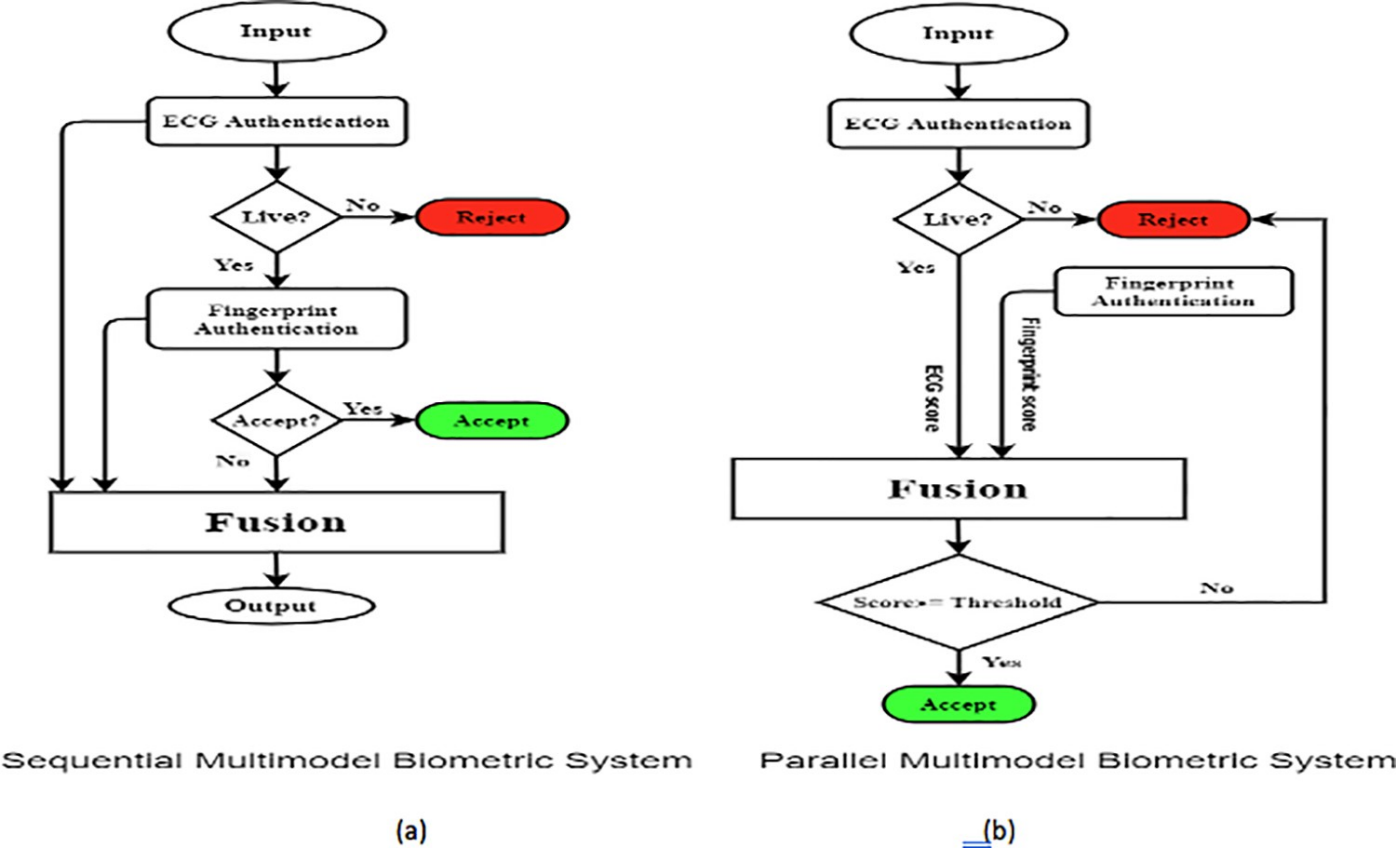
Multimodal biometrics system.

The block diagram of a sequential multimodal biometric system is shown in Fig 5A. The sequential multimodal structure depends on decision level fusion starts with ECG signal identification to guarantee that the accepted score is generated from an alive subject. Whereas ECG identification is better for rejecting impostors and fingerprint identification is better for accepting genuine subjects. Rejection is the final decision of the system if the subject is rejected according to its ECG. If the subject is accepted, it is fed to the multimodal biometric system to combine them at the decision level to produce the final decision. After the genuine acceptance and the impostors’ rejection by the ECG system, the other subjects are recognized by the decision level fusion of fingerprint and ECG. The combination of decision outputs is formed by applying (OR) logic rule to fulfill the greatest system performance. The block diagram of a parallel multimodal biometric system is demonstrated in Fig 5B. The parallel multimodal structure depends on score level fusion for fingerprint and ECG. The fingerprint and ECG of the subject are obtained in the verification stage, unlike the sequential multimodal system which does not require authenticating by both modalities. As features extraction is completed, the extracted features of both modalities are moved to classifiers for finding matching scores. Finally, a fusion rule is applied to the matching scores, where, ECG signal scores of accepted users are fused with their scores of the fingerprint to produce the final decision upon score level fusion. If the fused score < 90% (a predefined threshold), the user is rejected as an impostor, otherwise the user is accepted as genuine. In this study, Fuzzy Logic (FL), Linear Discriminant Analysis (LDA), or Neural Network (NN) is used for the fusion. One of these classification methods is executed to get the scores of each biometric. Subsequently, the fusion rule is applied to fingerprint scores and ECG scores to obtain the final score. One of three fusion rules that are Product, Sum (Summation), and (MAX) Maximum rules is applied. The applicable threshold is 90%, yet, BE et al. [51]set a low threshold (75%) in their proposed model.

#### Biometric models based on CNN

Recently, Deep learning is used in different domains especially CNN. The structure of CNN includes hidden layers and different parameters [57]. There are several CNN models like Caffe-Net, Alex-Net, and VGG-Net for large-scale image classification [21]. Where VGG-Net is utilized for feature extraction of fingerprint and ECG in the proposed CNN model because its architecture is much deeper than other models [57].

Simonyan and Zisserman [59] proposed the VGG-Net to deepen the network and enhance classifier performance by integrating multiple convolutional layers. This may be accomplished by using several stacked kernel filters in place of a large sized filter. Every few layers, the max pooling layer is utilized to minimize the spatial dimensions. The first network structure to use a block-based architecture is the VGG. All hidden layers now include ReLU nonlinearity. VGG has more weight parameters than AlexNet, but it converges faster because of taking fewer epochs due to the regularization implication by its depth and the small size of convolution filter [60]. More details in [59].

**Fig 6:**
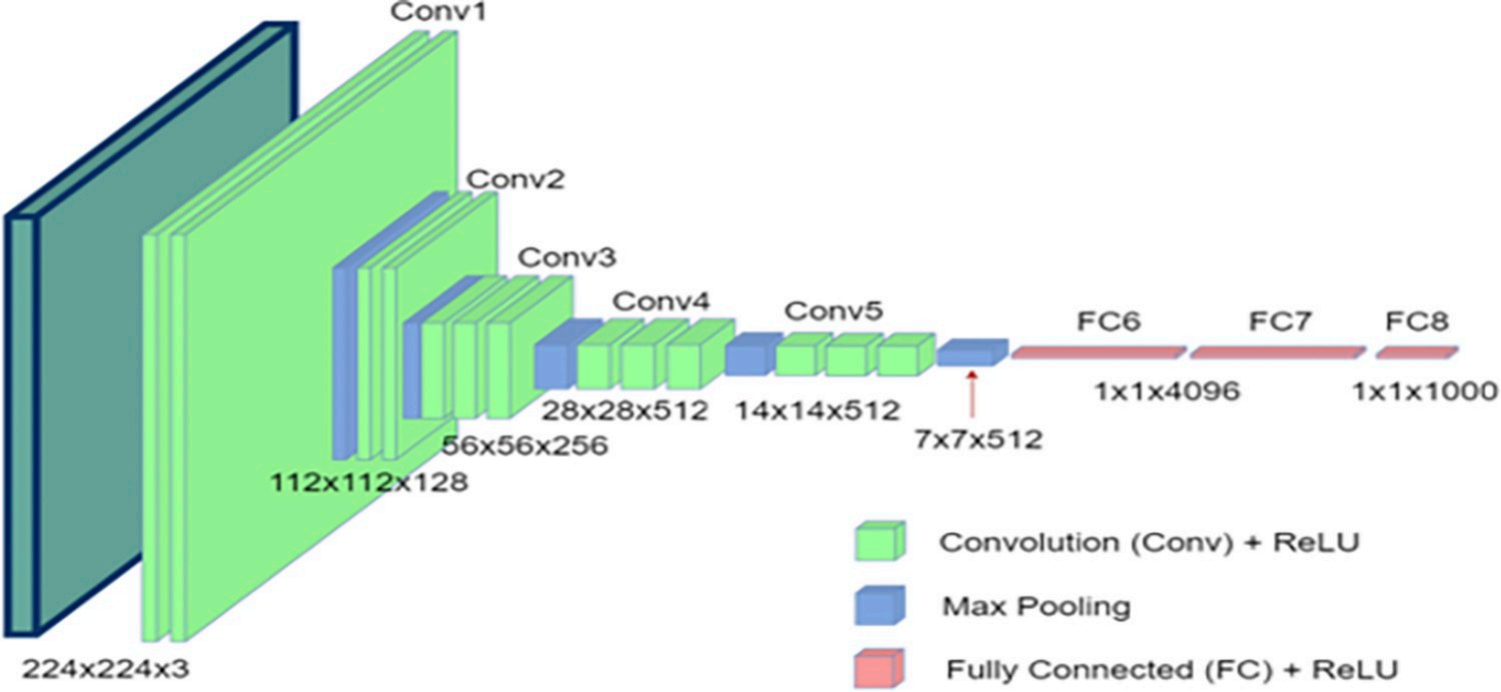
VGG-Net architecture.

In this study, the VGG-Net architecture is indicated in Fig 6. It involves five convolutional (Conv) layers which are followed by a pooling layer for each and 3 FC (fully connected) layers and Fig 6 shows all the parameters that are used in this work. The activation function is Rectified Linear Unit (ReLU) in all hidden layers.

The features that obtained from both ECG and fingerprint modalities are further processed based on a fusion module as indicated in Fig 8. Where, the fingerprint feature vector is concatenated into an ECG feature vector to generate a single features vector. The concatenated feature is entered an additional fully connected layer to determine the final class.

#### ECG biometric system based on CNN

ECG-CNN recognition system using a CNN model unlike ECG recognition system using the traditional methods, where it used ECG signals without noise correction of segmentation methods. The main phases of ECG-CNN include the transformation of ECG signals (1-D) into ECG images (2-D) by plotting each in a 224×224 grayscale image as in [57]. Consequently, 2-D CNN can be applied to ECG images. Then, the features template is formed by the ECG-CNN model before the classification phase.

#### Fingerprint biometric system based on CNN

The feature extraction from fingerprint images in FP-CNN recognition system is achieved by VGG-Net. The main phases of FP-CNN include preprocessing, the extraction of features using VGG-Net, and the classification phase.

### Multimodal systems based on CNN

#### Sequential multimodal system based on CNN

This system is a sequential multimodal biometric system with CNN and the decision level fusion of fingerprint (FP-CNN) and ECG signal (ECG-CNN) for subject identification. The extraction of modalities features is achieved by utilizing CNN and these features formed the biometric templates. Then, the classification techniques are applied to enhance the system performance and the decision level fusion is applied for making the decision. This system according to the decision level fusion begins with ECG signal identification to maintain accepted score generated from an alive subject. Whereas, ECG identification is suitable for rejecting impostors and fingerprint identification is better for accepting genuine individuals. Fig 5 explains the sequential multimodal biometric system. Rejection is the final decision of the overall system if the subject is rejected due to its ECG. Yet, If the subject is accepted, it is fed to the multimodal system to combine them at the level of decision to produce the final decision. After the genuine acceptance and the impostors’ rejection by the ECG model, the other individuals are identified by the decision fusion of fingerprint and ECG. The combination of decision outputs is formed by OR Rule to fulfill the best system performance.

#### Parallel multimodal system based on CNN

**Fig 7:**
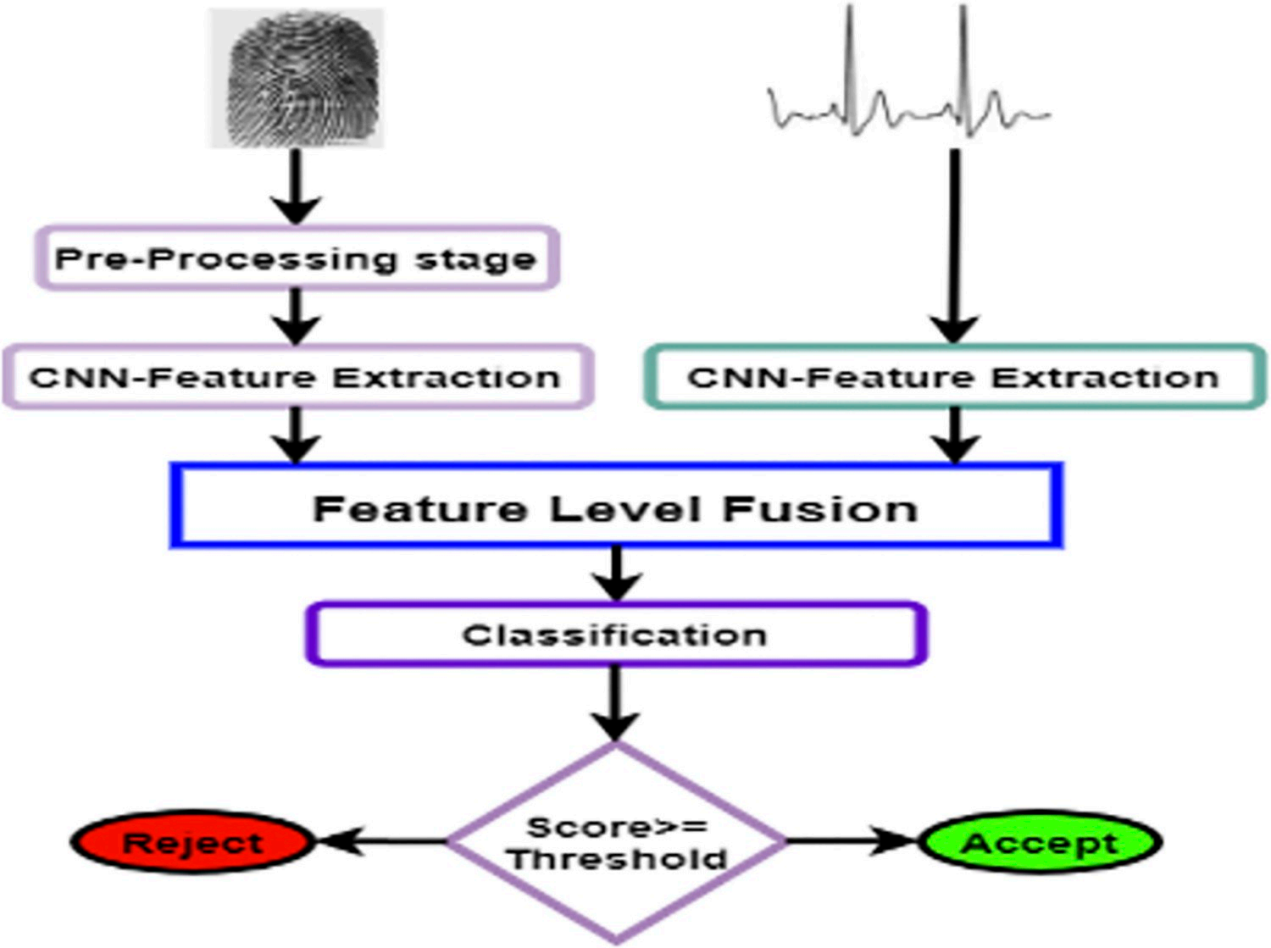
Parallel multimodal biometric system.

**Fig 8:**
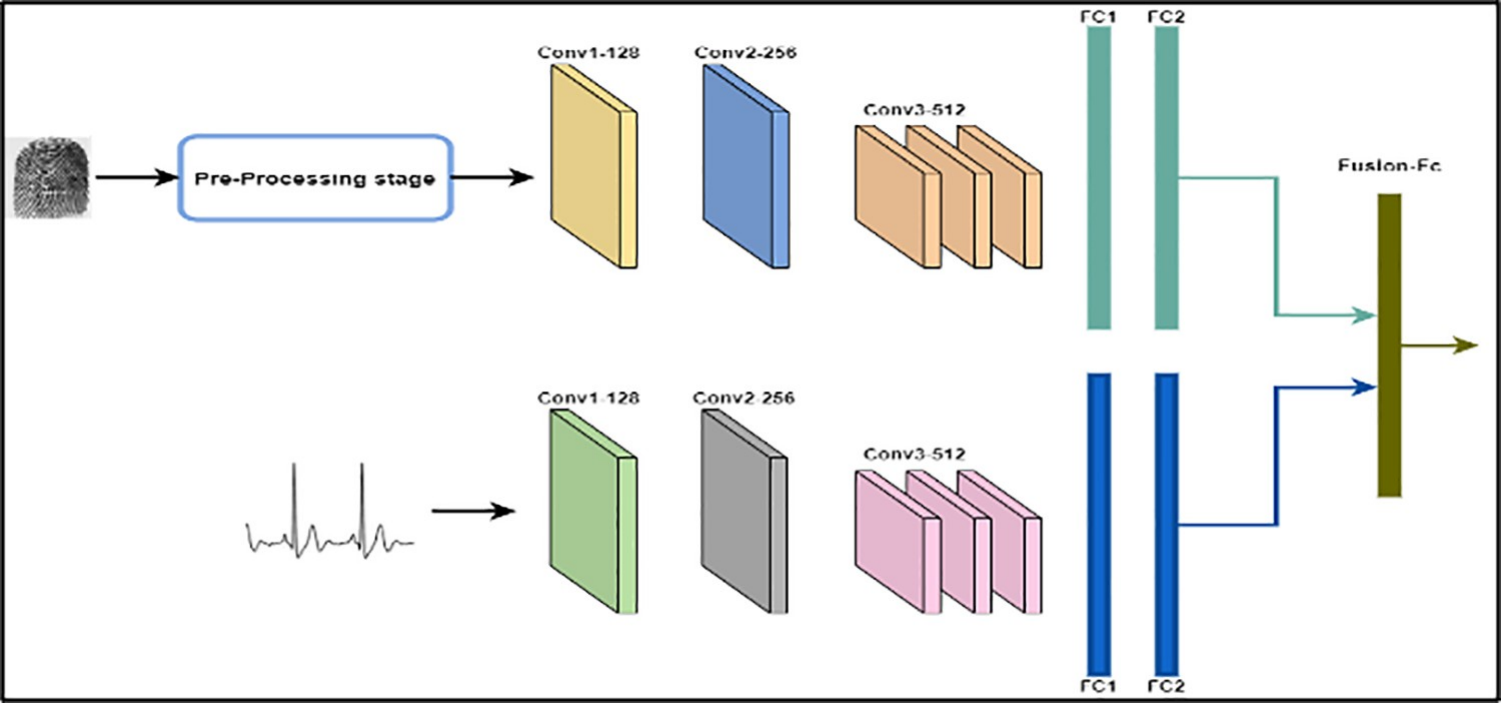
Multimodal biometric system depend on multi-CNN features fusion.

This system is a parallel multimodal biometric system utilizing CNN and the feature fusion of fingerprint and ECG signal for subject identification. The extraction of modalities features is achieved by utilizing CNN and the resulting feature vectors are combined using the fusion level method. The parallel multimodal system with CNN for fingerprint and ECG is shown in Fig 7. The fingerprint and ECG of the subject are presented in the verification stage, unlike the sequential multimodal that does not require authenticating by two modalities. The extracting feature of specific modalities is achieved by the same CNN utilized in the sequential multimodal. The extracted feature vectors of both modalities are applied to the feature level fusion before the classification as indicated in Fig 8.

## Results and discussion

### Performance measurements

There are various metrics to measure the system performance for evaluation, which are described below [16]:

■ **ROC Curve:** is a graphical representation which describes the performance with classification accuracy [12].
■ **Area Under the ROC Curve (AUC):** computes total 2-dimensional area below the ROC curve, as AUC indicates better performance when it is increased near to 1 [12].
■ **Specificity (True Negative Rate)**: computes negatives proportion correctly identified as in Eq (4) [17]:
$$Specificity=True\;Negative\;Rate=\frac{TN}{TN+FP}$$ (4)
  where *FP* is False Positive that represent number of imposter acceptance, and *TN* is True Negative that represent imposter rejection number [17, 56, 57].
■ **Sensitivity (True Positive Rate)**: computes positives proportion correctly identified as in Eq (5) [16]:
$$Sensitivity=True\;Positive\;Rate=\frac{TP}{TP+FN}$$ (5)
  where FN is False Negative which represents legitimate rejection number, and TP is True Positive that represent legitimate acceptances number [17, 56, 57].
■ **Efficiency:** produces the ratio of times where test produces correct result to overall number of tests as in Eq (6) [17]:
$$Efficiency=\frac{TP+TN}{TP+TN+FP+FN}$$ (6)

### Simulation results

#### Fingerprint biometric recognition system

**Fig 9:**
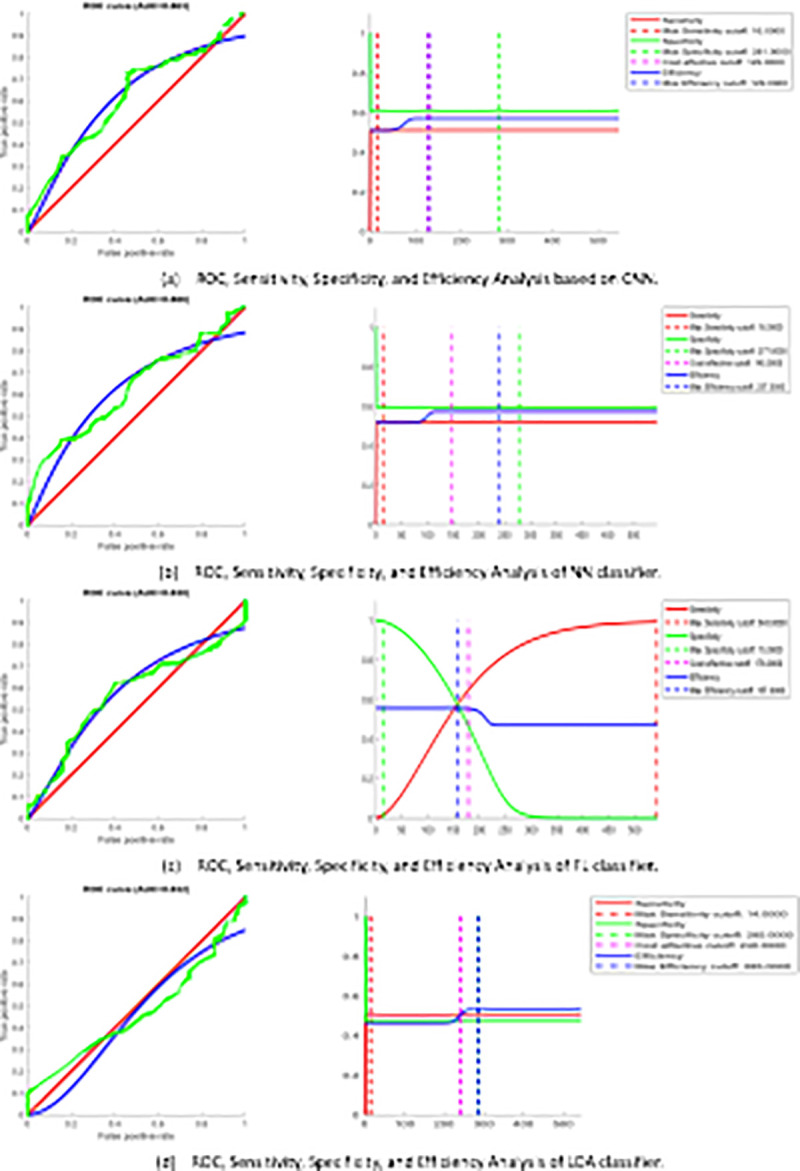
ROC curve evaluation of fingerprint recognition system.

The fingerprint recognition system is examined using the FVC2004 database (FVC2004) with a ten-fold cross-validation algorithm. Findings (AUC %) for fingerprint recognition system are (CNN = 86.3, NN = 86.6, FL = 83, LDA = 83.2). The performance results of proposed system are reported in Table 4 and while Fig 9 shows the ROC curve for fingerprint recognition system. The presented system implies a comparable performance of other good systems. Generally, the suggested system signifies an acceptable performance with more robust results, reliable and effective computational time and computational cost compared to the works cited in Table 1.

**Table 4:** Performance of fingerprint recognition system.

Fingerprint Recognition System	AUC	Standardized AUC	SEM	CI
Based on CNN	86.3	39.1574	0.00928	0.86276–0.86392
Based on NN Classifier	86.6	41.7123	0.00879	0.86590–0.86700
Based on FL Classifier	83	28.7231	0.01148	0.82895–0.83039
Based on LDA Classifier	83.2	13.4341	0.01470	0.69654–0.69838

#### ECG biometric recognition system

**Fig 10:**
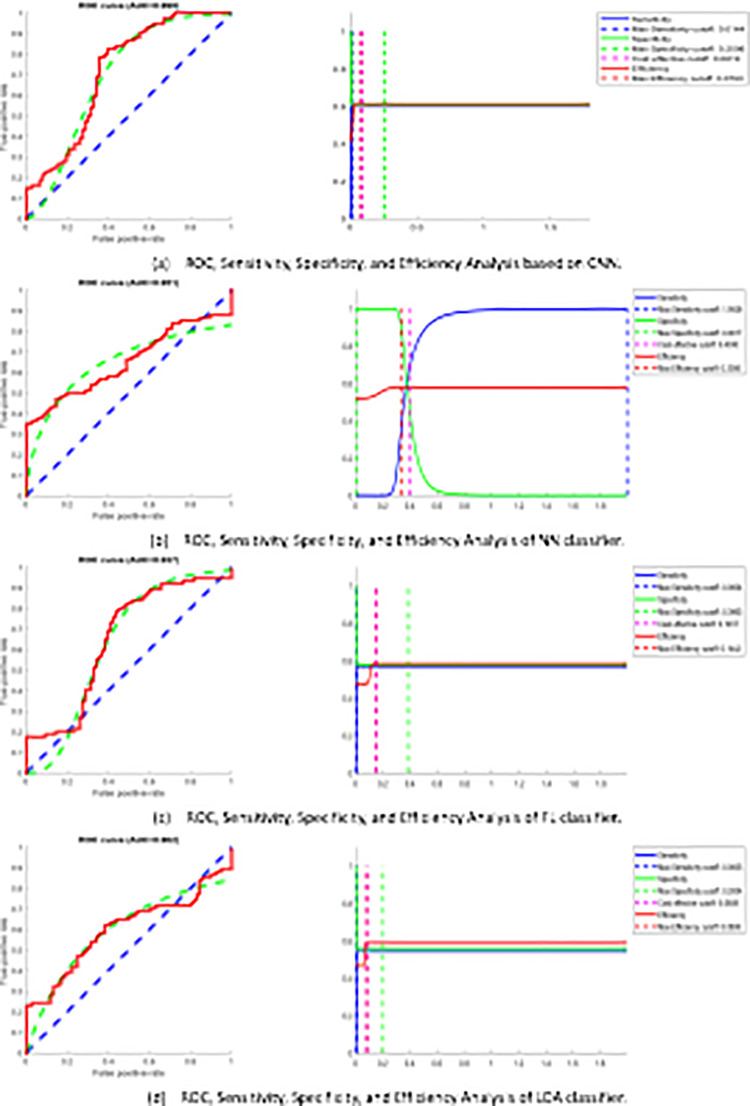
ROC curve evaluation of ECG recognition system.

The implementation of the ECG recognition system is examined using 47 subjects MIT-BIH database [54], utilizing ten-fold cross-validation algorithm, to explain, ECG signals are divided into ten equal folds, nine folds are used for training while the residual is used for testing. The metrics (AUC %) for the ECG recognition system are (CNN = 99.863, NN = 95.1, FL = 93.688, LDA = 90.214). The performance results of proposed system are reported in Table 5 and while Fig 10 shows the ROC curve for the ECG recognition system. The suggested system signifies a comparable performance of other good systems. Generally, the suggested system shows better performance with more robust results compared to the works cited in Table 2.

**Table 5:** Performance of ECG recognition system.

ECG Recognition System	AUC	Standardized AUC	SEM	CI
Based on CNN	99.863	162.8990	0.00306	0.99805–0.99921
Based on NN Classifier	95.1	24.4400	0.01845	0.94738–0.95436
Based on FL Classifier	936.88	20.8867	0.02092	0.93293–0.94084
Based on LDA Classifier	90.214	15.4642	0.02600	0.89722–0.9070

### ECG and fingerprint multimodal biometric systems

#### Proposed sequential multimodal system

**Fig 11:**
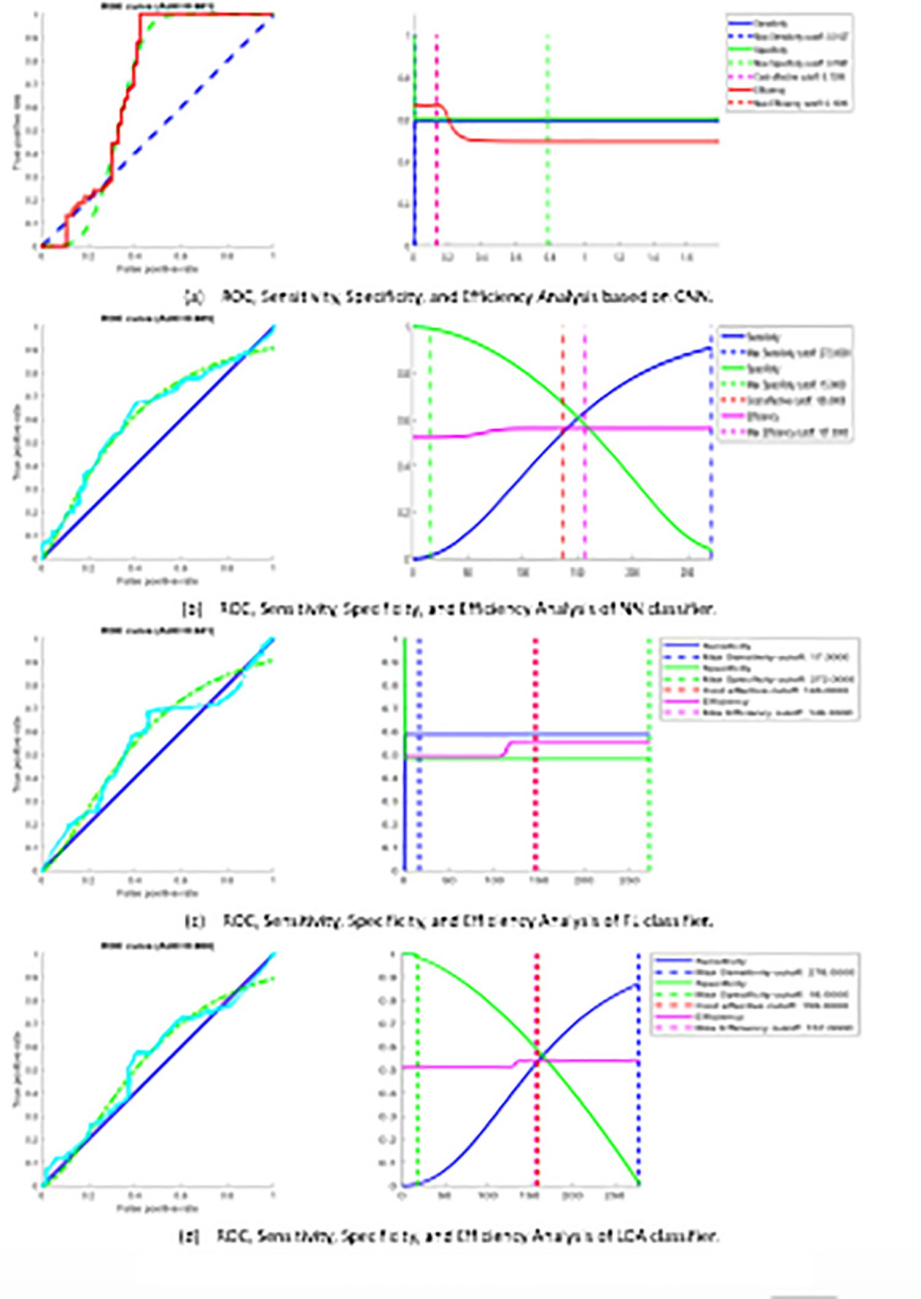
ROC curve analysis of sequential multimodal system.

The virtual multi-modal dataset is used in the assessment of the suggested parallel multi-modal system according to the decision level fusion. The metrics (AUC %) for sequential multimodal biometric recognition system are (CNN = 98.1, NN = 98.5, FL = 94.1, LDA = 90.8). The performance results of proposed system are reported in Table 6 as well as ROC curves for multimodal biometric recognition system based on 3 different fusion rules at threshold 90% are shown in Fig 11.

**Table 6:** Performance of sequential multimodal system.

Sequential Multimodal System	AUC	Standardized AUC	SEM	CI
**Based on CNN**	98.1	41.6712	0.01154	0.97851–0.98287
**Based on NN Classifier**	98.5	139.4800	0.00347	0.98456–0.98465
**Based on FL Classifier**	94.1	75.2698	0.00586	0.94130–0.94144
**Based on LDA Classifier**	90.8	49.5193	0.00824	0.90807–0.90828

#### Proposed parallel multimodal system

**Fig 12:**
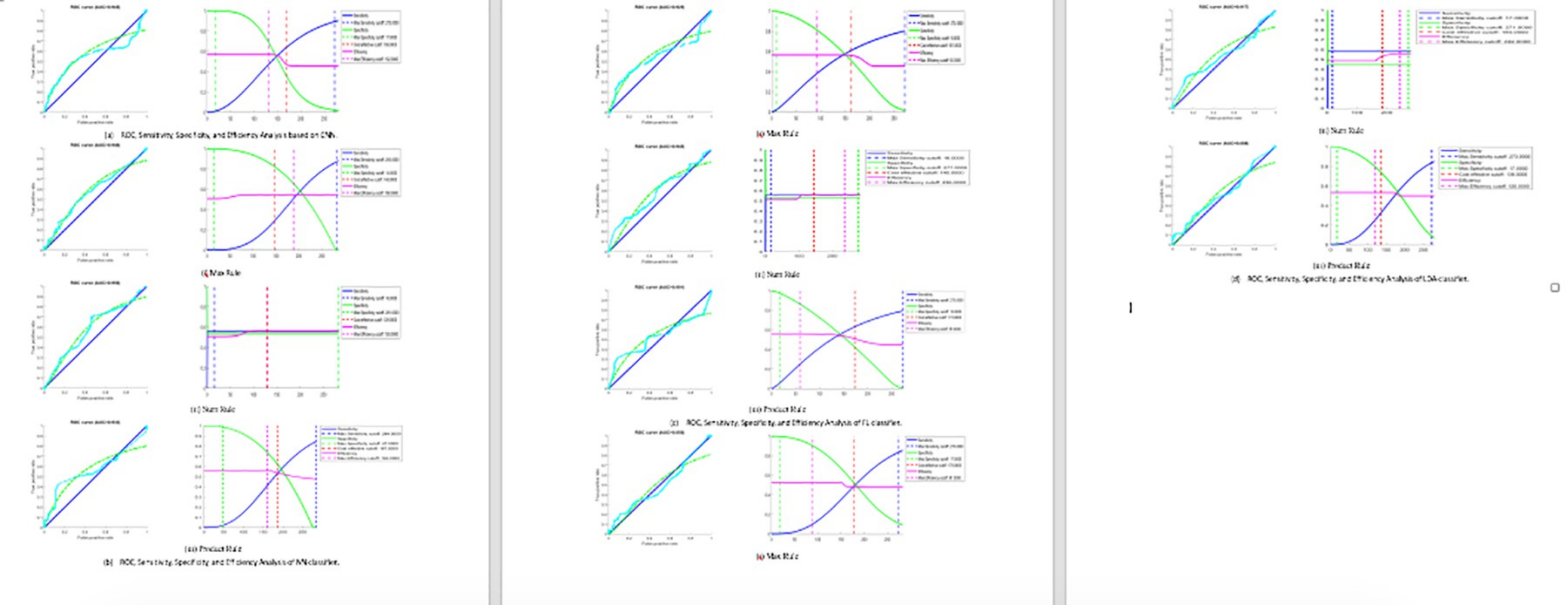
ROC curve analysis of parallel multimodal system.

The virtual multi-modal dataset is used in the evaluation of the proposed parallel multi-modal approach according to the score level fusion. The metrics for parallel multimodal biometric recognition system are stated in Table 7 as well as ROC curves for multimodal biometric recognition system based on 3 different fusion rules for each classifier at threshold 90% are shown in Fig 12.

**Table 7:** Performance of parallel multimodal system.

Parallel Multimodal System	Based on CNN	Based on NN Classifier	Based on FL Classifier	Based on LDA Classifier
**AUC(%)**	94.5	Max Rule = 94.6	Max Rule = 92.5	Max Rule = 85.6
		Sum Rule = 95.6	Sum Rule = 94.5	Sum Rule = 91.7
		Product Rule = 93.3	Product Rule = 90.1	Product Rule = 88.8

#### Simulation results based on data augmentation

The dataset augmentation impact on the proposed multimodal system is indicated in Table 8. The dataset augmentation impact in terms of the error reduction on the suggested multimodal system is 0.01845 without augmentation and 0.003 with augmentation. The average error of the proposed system without augmentation is greater than the average error with augmentation. So, the accuracy of the proposed system with augmentation has been improved.

**Table 8:** Dataset augmentation impact.

Without augmentation	With augmentation
0.01845	0.003

#### Comparison between the suggested approach and other systems

According to this study, the performance of ECG and fingerprint fusion biometric modalities with different classification techniques and different fusion levels is evaluated. By comparing with existing systems, the proposed unimodal (ECG recognition system & fingerprint recognition systems) and multimodal systems have achieved acceptable recognition results. Overall, this work has a good contribution and has presented sufficient experimental results.

**Fig 13:**
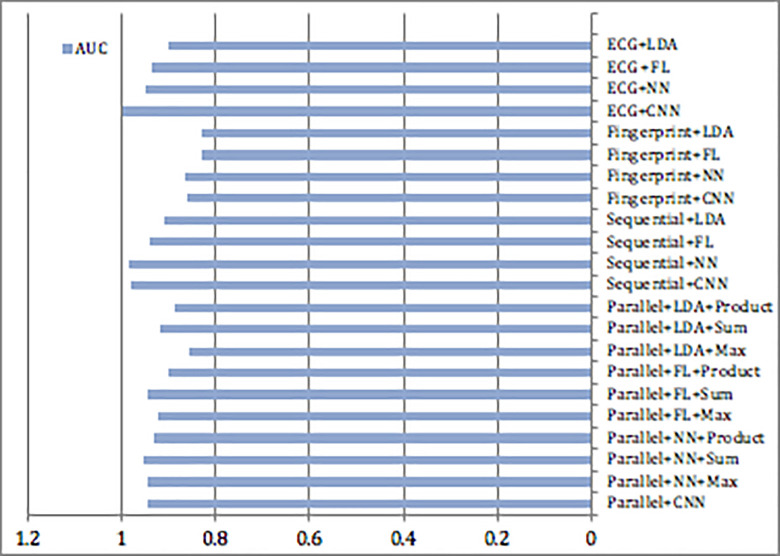
Performance measurements comparison of proposed systems.

The comparison between the unimodal and multimodal based on traditional and deep learning models is indicated in Fig 13. Most of the research either focused on certain features and artificial intelligence algorithms for identification or did not incorporate fusion levels to produce accurate results. Measurement of accuracy depending on the features used are reported. Because there is no complete database to test the approach, the data was combined as described in section “Dataset Information” and it is widely approach for this type of research. The approaches proposed by [42, 51, 57, 56] are authentication techniques that are combination of (ECG) and fingerprint multimodal. Some of the proposed systems suggested to test model on a real database. [21, 38, 47] have used face and other biometric traits in biometric identification systems and obtained better results than using two or one biometric traits. [52, 53] used ECG, Face, and Fingerprint for authentication, these multibiometric system fusing the ECG signal with the face and fingerprint biometrics. Yudistira & Kurita [24] Suggest a correlation network with a Shannon fusion for learning a pre-trained CNN it also emphasizes the importance of multimodal correlation as it enhanced the accuracy of the recognition results. As most of research relies on classical algorithms and few were dependent on deep learning, we propose the novelty in employing advanced deep learning into multi and single modalities with different fusion levels. For effectiveness of the approach, we utilize the known metrics in classification as each scholar presented different metrics depending on their method. Thus, even if different metrics used, the usability and reliability of the system can be implied. Generally, the suggested multimodal system implies an acceptable performance, signifies a similar performance of other good systems, and is more robust than previous works covered in Tables 3 and 9. So, the suggested multimodal systems can be adopted for recognition with adequate performance.

**Table 9:** Comparison between the suggested approach and other systems.

Researchers	Modalities	Sample Size + Dataset	Fusion Techniques	Performance Measurements
[51]	ECG and Fingerprint	Biopac is used for ECG signal, MP35 for different subjects	Score Generation	ECG: Matching Rate up to 69% when FAR = 2.38, FRR = 9.52 Fingerprint: Matching Rate up to 88.89% when FAR = 7.77, FRR = 5.55 Multimodal Biometric: Matching Rate up to 92.8% when FAR = 2.5, FRR = 0
[56]	ECG and fingerprint	200 Subjects (PTB + LivDet2015)	CNN (Parallel score fusion)	Finger: Acc = 98.48% ECG: Acc: 96.56% FAR: 0.033% FRR: 0% Multimodal: ◾ Sum: Acc 99.99 ◾ Max: Acc 99.50 Product: Acc 95.16
[57]	ECG and fingerprint	ECG: PTB(100) CYBHi(63) Fingerprint: FVC 2004 LivDet2015	CNN (Q-Gaussian multi support vector machine)	ECG-PTB: ◾ without internal fusion: Acc 96.83% ◾ internal fusion (concatenation): Acc 97.50% ◾ fusion (addition): Acc of 98.66% ECG-CYBHi: ◾ without fusion: Acc 97.15% ◾ fusion (concatenation): Acc 98.44% ◾ fusion (addition): Acc of 98.97% Finger- LivDet2015: ◾ without fusion: Acc 97.12% ◾ fusion (concatenation): Acc 98.25% ◾ fusion (addition): Acc of 98.81% Finger- FVC 2004: ◾ without fusion: Acc 96.70% ◾ fusion (concatenation): Acc 97.40% ◾ fusion (addition): Acc of 98.20% Multimodal: MDB1: ◾ without fusion: Acc 99.12% ◾ fusion (concatenation): Acc 99.55% ◾ fusion (addition): Acc of 99.83% MDB2: ◾ without fusion: Acc 99.42% ◾ fusion (concatenation): Acc 99.82% fusion (addition): Acc of 99.92%
[21]	face, finger vein, and iris	SDUMLA-HMT dataset	CNN + score and feature levels	Acc = 99.39% with a feature level Acc = 100% with score level
[24]	spatial and temporal streams	UCF-101 + HMDB-51 datasets	CNN+ Shannon fusion approach	Acc = 94.2
[47]	Face and Iris	CASIA-Face V5 + IITD iris datasets	CNN	Findings proved the superiority of suggested multimodal system compared with unimodal one.
[38]	Fingerprint, Face, and Hand Geometry	100 Subjects	Min-score Max-score Sum of scores	Fingerprint: Acceptance Rate nearly 83.6% Multibiometric: ○ Min-score: 85.6% ○ Max-score: 93.6% ○ Sum of scores: 98.6%
[42]	ECG and Fingerprint	45 Subject	SVM Weighted Sum Rule Likelihood Ratio	ECG: EER = 6.97% Fingerprint: EER = 2.22% Multimodal: EER = 1%
[52]	ECG, Face, and Fingerprint	50 Subjects	Weighted Sum Rule. Likelihood SVM	Multimodal outperforms unimodal biometrics system.
[53]	ECG, face, and fingerprint	78 Subjects	score fusion with Transformation	Multi-biometric system: EER = 0.22% ECG: EER = 10.80% Face: EER = 4.52% Finger: EER = 2.12%,.
Proposed	ECG and fingerprint	47 Subjects	CNN FL NN LDA	Parallel+CNN 94.5 (AUC%) Parallel+NN+Max 94.6 Parallel+NN+Sum 95.6 Parallel+NN+Product 93.3 Parallel+FL+Max 92.5 Parallel+FL+Sum 94.5 Parallel+FL+Product 90.1 Parallel+LDA+Max 85.6 Parallel+LDA+Sum 91.7 Parallel+LDA+Product 88.8 Sequential+CNN 98.1 Sequential+NN 98.5 Sequential+FL 94.1 Sequential+LDA 90.8 Fingerprint+CNN 86.3 Fingerprint+NN 86.6 Fingerprint+FL 83 Fingerprint+LDA 83.2 ECG+CNN 99.863 ECG+NN 95.1 ECG +FL 93.688 ECG+LDA 90.214

## Conclusion

A strong multiple biometric modalities can greatly enhance the security of a system. They provide anti-spoofing measurement, where the intruder will find it difficult to spoof multiple biometric modalities, ECG information combined with other biometrics will minimize credentials forgery and intrusion. A superior recognition performance with deep learning can be obtained in a multimodal biometric system at different fusion levels over unimodal technique. Utilizing ECG as one of the biometrics provides an advantage to the system where the heartbeat is inherited to a subject that is secured, confidential, and hard to be forged. In this study, unimodal and multimodal biometric systems using CNN are presented using different levels of fusion of fingerprint and ECG signal. The performances of CNN and different traditional classification techniques with unimodal and multimodal biometric systems using ECG and fingerprint have been studied. A powerful data combination that is not easily guessed can increase the security of the system and reduce marginal error. The tests are performed on ECG and fingerprint records to assess the performance of the suggested multimodal biometric system. FVC2004 database is utilized for the fingerprint, MIT-BIH database is utilized for ECG, and other experiments are being implemented to assess the suggested multimodal system with 47 subjects using a virtual multimodal dataset. Further tests are being conducted to evaluate the proposed models with ODB and VDB datasets.

The suggested CNN model for ECG signal and sequential multimodal system based on neural network outperformed other systems while optimum performance of the parallel multimodal achieved 0.96 Area Under the ROC Curve (AUC) and sequential multimodal achieved 0.99 AUC, in comparison to unimodal biometrics which achieved 0.87 and 0.99 AUCs, for the fingerprint and ECG biometrics. The performance of the proposed multimodal biometrics outperformed unimodal biometrics using CNN. Generally, the results imply that the suggested unimodal and multimodal biometric systems based on ECG and fingerprint using deep learning have satisfactory identification results compared to other previous works.
